# Complete mitochondrial genome sequence of tufted deer (*Elaphodus cephalophus*) with phylogenetic analysis

**DOI:** 10.1080/23802359.2019.1673235

**Published:** 2019-10-25

**Authors:** Lin Zhang, Yongfang Yao, Diyan Li, Jiayun Wu, Anxiang Wen, Meng Xie, Qin Wang, Guangxiang Zhu, Qingyong Ni, Mingwang Zhang, Huailiang Xu

**Affiliations:** a College of Life Science, Sichuan Agricultural University, Ya’an, China;; b College of Animal Science and Technology, Sichuan Agricultural University, Chengdu, China

**Keywords:** Mitochondrial genome, *Elaphodus cephalophus*, phylogenetic analysis

## Abstract

The complete mitochondrial genome of tufted deer (*Elaphodus cephalophus*) has been described in our research. The sequence of this genome is 16356 bp with a circular structure, and contains 13 protein-coding genes (PCGs), 22 transfer RNA (tRNA) genes, two ribosomal RNA genes, and one control region. When compare with the reference sequence (DQ873526), it shows that there are 262 variations, 4 base deletions and 13 base insertions. In this study we obtain a new complete mitochondrial genome sequence of tufted deer that provide effective molecular information for the diversity research and phylogenetic structure.

Tufted deer (*Elaphodus cephalophus*), one kind of solitary dark-brown small herbivore with a tuft of hair on its crown and sharp canines, always lives in moist mountain forests within well-defined home territories at the elevations of 300–4750 meters from southeast to southwest of China and probably in northwestern Myanmar according to historical records (Geist 1998; Francis 2008; Smith and Xie 2008; Wilson and Mittermeier 2011). This species is listed as ‘‘Near Threatened’’ by the International Union for Conservation of Nature (IUCN) and thought to be on the verge of a significant population decline due to overhunting that would make it close to qualifying for vulnerable under criterion A2d (Sheng and Lu 1985; Ohtaishi and Gao 1990; Grubb 2005). Furthermore, there is little research on the species of the tufted deer and poorly is known about its genetic diversity, so it is necessary to determine its current status in the wild (Sheng et al. 1998; IUCN 2015). We obtained a complete mitochondrial genome sequence from the tufted deer muscle tissue, which was preserved in the museum of sichuan agricultural university (Accession: 000753) that was killed by illegal poaching in Yingjing county (N29°30′, E102°30′), Sichuan, China, in order to provide effective molecular information for the research and classification status of tufted deer.

The total genomic DNA was extracted with the TIANamp Genomic DNA Kit. Fourteen pairs of primer were designed base on the reference sequence (DQ873526), which download from the Genbank. We amplify the mitochondrial genome of tufted deer successfully and acquire a sequence of complete mitochondrial genome of tufted deer.

We sequenced the complete genome of the mitochondria of this species and obtained a 16356 bp long sequence (Genbank number MN251783), which contains thirteen protein-coding genes (PCGs), twenty-two transfer RNA (tRNA) genes, two ribosomal RNA (rRNA) genes, and one control region (D-loop) that most were similar to vertebrates (Roos 2018). There is only one gene (*ND6*) in PCGs and eight tRNA genes (tRNA^Gln^, tRNA^Ala^, tRNA^Asn^, tRNA^Cys^, tRNA^Tyr^, tRNA^Ser^, tRNA^Glu^, tRNA^Pro^) encoded on the L-strand. About PCGs, all the other initiation codon is ATG except *ND2*, *ND3*, *ND4L*, *ND5* and *ND6*, and most termination codon are TAG and TAA except some incomplete stop codon (TA– or T–). The base composition of mitochondrial DNA in Tufted deer is A(33.26%), T(29.19%), C(24.11%), G(13.44%), and the percentage of A + T (62.45%) is higher than C + G (37.55%), it shows a bias in A\T. Comparing the sequence (MN251783) to the reference sequence (DQ873526) shows that there are 262 base mutation sites, four base deletion sites and 13 base insertion sites.

A Maximum-likelihood (ML) phylogenetic tree with fourteen species complete mitochondrial genome of cervidae and one outgroup (*Moschus berezovskii*) was constructed in MEGA10.0 (Kumar et al. 2018), in order to further explore the phylogenetic analysis of tufted deer (Figure 1). The ML phylogenetic tree provided strongly support for the classification of our samples. Our study revealed a unique complete mitochondrial genome sequence of tufted deer that can be used for molecular identification and species classification.

**Figure 1.**
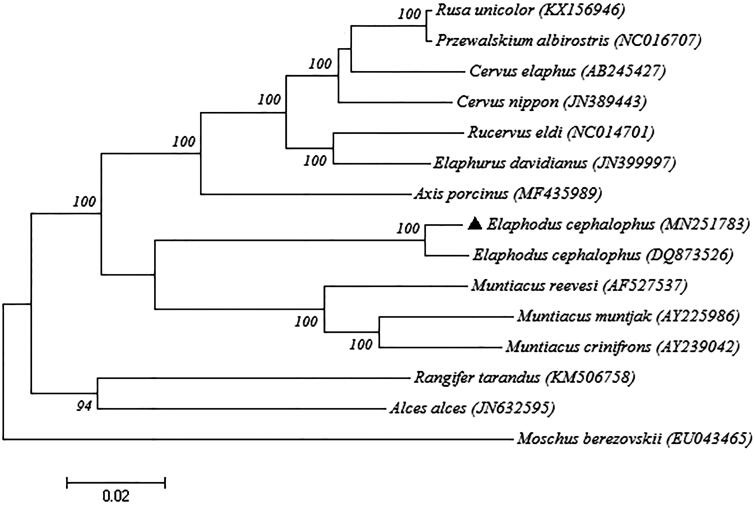
Maximum-likelihood phylogenetic tree constructed based on fourteen species complete mitochondrial genome of cervidae and one outgroup (*Moschus berezovskii*). Numbers on nodes refer to bootstrap values and GenBank accession numbers are listed below species. Elaphodus cephalophus represented a sequence in this study.
